# Association of Hemodialysis Inadequacy and Duration with Restless Legs Syndrome: A Cross-sectional Study

**DOI:** 10.7759/cureus.2570

**Published:** 2018-05-02

**Authors:** Zain Ul Abideen, Syed Nayer Mahmud, Fahad Mushtaq, Muhammad Umer Farooq, Yusaf Farooq Qasim, Zamara Hamid, Amna Rasheed, Muhammad Zafran, Muhammad Nabeel Pasha, Faiqa Pirzada

**Affiliations:** 1 Nephrology and Renal Transplant, Royal Cornwall Hospital; 2 Department of Nephrology, Shifa International Hospital, Islamabad, Pakistan, Islamabad , PAK; 3 Shifa College of Medicine, Shifa Tameer-e-Millat University; 4 Medicine, Shifa International Hospital, Islamabad, PAK; 5 Department of Internal Medicine, Shifa International Hospital, Islamabad, PAK; 6 Respiratory Department , Royal Cornwall Hospital, Royal Cornwall Hospital, Truro, GBR; 7 Pediatrics, Bahawal Victoria Hospital Bahawalpur

**Keywords:** restless legs syndrome, hemodialysis patients

## Abstract

Restless legs syndrome (RLS) is a common neurological disorder in hemodialysis (HD) patients. It is associated with poor sleep and decreased quality of life. The precipitants for the disorder are still poorly understood. The condition has not been studied extensively in Pakistan, which has a vast majority of end-stage renal disease patients on maintenance HD. We aimed to determine the prevalence of this condition in patients attending HD units of the largest renal dialysis center in Northern Pakistan. We also strived to determine any associations with dialysis inadequacy and the total duration of HD. This was an observational study comprising 279 patients. RLS was diagnosed using the International Restless Leg Syndrome Study Group criteria. Dialysis adequacy was determined using the Urea Reduction Ratio and the Kt/V technique. The prevalence of RLS in this large HD population was 24%. Our results show that a longer duration and greater number of HD sessions were significantly associated with the development of RLS (p<0.05). Dialysis inadequacy was not associated with the development of the disorder. These results may indicate that the pro-inflammatory nature of hemodialysis may have a role in the pathophysiology of RLS in HD patients and prolonged exposure to it may make them more prone to developing the disorder.

## Introduction

Restless legs syndrome (RLS), also known as Willis-Ekbom disease, is a neurological disorder that has a detrimental effect on the patient's quality of life. Symptoms are usually present in the legs, but may also involve other areas of the body [1]. The patient may describe a strong desire to move the limbs, a feeling of something crawling on the skin, an itching sensation or other sensorineural symptoms. Symptoms tend to peak at night, which makes it difficult for the patient to sleep [2]. The condition is grossly under-reported, with a prevalence ranging between 5-15% in the general population, and being more common in women [3-5]. While not completely understood, the pathophysiology of Willis-Ekbom disease is probably related to decreased iron reserves, improper iron regulation, and dopamine imbalances within the central and peripheral nervous systems [6]. The prevalence of RLS in patients on hemodialysis (HD) is higher than in the general population. Studies report prevalence between 20-30% [7,8]. In Pakistan, RLS in patients on hemodialysis has not been extensively studied. A few studies have been done, which report a prevalence of 20-65%. This large variation is due to varying sample sizes and different RLS diagnostic criteria [9-11]. Dialysis inadequacy is a predictor of mortality in dialysis patients [1]. Past studies do not associate dialysis inadequacy with RLS; however, this has not been properly studied in the Pakistani population. The fact that symptoms of RLS abate after renal transplantation also raises a question as to whether prolonged exposure to HD predisposes to RLS. Results regarding this have been conflicting in previous studies. Therefore with these questions in mind, our aim was to ascertain the prevalence of RLS in the HD population at Shifa International Hospital, which is the largest HD unit in Northern Pakistan. We also aimed to determine any association between RLS and inadequacy/duration of HD.

## Materials and methods

We conducted an observational cross-sectional study at two HD units of Shifa International Hospital (SIH) Islamabad over a period of four months. We used consecutive non-probability sampling to include patients in our study. Approval for conducting the study was sought from the Institutional Review Board and Ethics Committee of SIH. Data collection was performed from January 2017 to April 2017.

All stable and mentally competent adults aged 18 years and above with end-stage renal disease (ESRD) and maintained on HD for at least six months were offered participation in the study after informed consent. Patients who were confused or had dementia were not approached. Patients with iron deficiency, Parkinson’s disease, peripheral neuropathy, lower limb lymphedema, deep vein thrombosis, arthritis, and lower limb cellulitis were excluded. Patients diagnosed with RLS before starting HD were also excluded.

Data collection was carried out by personal interview, using the standardized diagnostic questionnaire endorsed by the International Restless Legs Syndrome Study Group (IRLSSG) [12]. This was delivered to patients in a local language (Urdu) and was pilot tested successfully before the data collection. Patients were evaluated by face-to-face interviews. All members of the team rehearsed the interview in regular meetings to ensure uniform and similar delivery of information to the patients to reduce bias. Written informed consent was taken from all participants and confidentiality of their data assured. The purpose of the study and proposed use of the findings was also explained to them.

The IRLSSG criteria are detailed below (all must be met for a diagnosis)

1. An urge to move the legs usually but not always accompanied by or felt to be caused by uncomfortable and unpleasant sensations in the legs.

2. The urge to move the legs and any accompanying unpleasant sensations begin or worsen during periods of rest or inactivity such as lying down or sitting.

3. The urge to move the legs and any accompanying unpleasant sensations are partially or totally relieved by movement, such as walking or stretching, at least as long as the activity continues.

4. The urge to move the legs and any accompanying unpleasant sensations during rest or inactivity only occur or are worse in the evening or night than during the day.

5. The occurrence of the above features is not solely accounted for as symptoms primary to another medical or a behavioral condition (e.g., myalgia, venous stasis, leg edema, arthritis, leg cramps, positional discomfort, habitual foot tapping).

The presence or absence of diabetes mellitus was noted. The most recent (less than a week) pre- and postdialysis blood urea levels were documented and the urea reduction ratio (URR) was calculated. The formula used was as follows:

URR = (Predialysis Urea – Postdialysis Urea / Predialysis Urea) × 100 = expressed as a percentage 

The cut-off values for URR used to determine inadequacy were <65% for patients dialyzing thrice a week and <80% for those dialyzing twice a week [13].

The other method used to determine dialysis adequacy was the Kt/V technique. K stands for the dialyzer clearance in ml/minute, which was noted for every patient, t stands for the time duration of dialysis in minutes, and V for the volume of water a patient’s body contains [14]. The latter was calculated by multiplying the patient’s body weight by 0.6 in males and 0.5 in females (takes into account that 60% of a male’s body weight and 50% of a female’s body weight is water) [13]. The average length of dialysis sessions was determined and the total duration of maintenance HD was determined in months. The frequency of dialysis sessions per week was also determined.

Data was entered and analyzed using Statistical Package for the Social Sciences (SPSS) version 23 (IBM Corp., Armonk, NY). Categorical variables were expressed as frequencies and percentages, while continuous variables as means and standard deviations. Categorical variables were compared using the chi-square test, while for continuous variables the Mann Whitney U test was employed. A p value of less than 0.05 was considered significant.

## Results

A total of 337 patients were approached; 279 were included and 58 were excluded. One hundred and forty-five patients (52%) were male, and 134 (48%) were females. The mean age of the patients was 53.34 ± 18.60 years. Fifty-eight patients (20.8%) underwent dialysis thrice a week while 220 (78.9%) dialyzed twice a week. One patient (0.4%) had dialysis once a week. The mean length of dialysis sessions was 3.9 ± 0.32 hours while the mean of duration on dialysis in months was 26.4 ± 22.78 months. The mean Kt/V was 1.2 ± 0.3, while the mean URR was 65.5 ± 15.3%. One hundred and three patients (36.9%) had diabetes mellitus while 176 (63.1%) did not have this disease.

The general characteristics of our study cohort are illustrated in Table 1.

**Table 1:** Characteristics of the hemodialysis study population (n=279) Abbreviations: URR - Urea Reduction Ratio; HD - Hemodialysis; TSAT- Transferrin saturation.

Variable	Value
Mean Age (years)	53.33 ± 18.59
Mean URR (%)	65.50 ± 15.30
Mean Kt/V	1.30 ± 0.15
Mean Serum Albumin (g/L)	36.00 ± 3.98
Mean Hemoglobin (g/L)	9.97 ± 1.60
Mean Serum Ferritin (ng/ml)	267±59
Mean TSAT (%)	24.0 ± 2.9
Mean Duration of HD Sessions (hours)	3.87 ± 0.32
Mean Number of Sessions	228.56 ± 188.76
Mean Number of Months on HD	26.41 ± 22.77
Male/Female	145(52%) /134 (48%)
Patients with Diabetes Mellitus	103/279 (36.9%)
Patients without Diabetes Mellitus	176/279 (63.1%)

Sixty-seven patients (24%) were diagnosed with RLS according to the above-mentioned criteria. Table 2 summarizes the difference between important variables in the RLS and non-RLS groups. On statistical analysis, total duration and total sessions on HD were significantly greater in the RLS than in the non-RLS group (p<0.05). The mean age was significantly different amongst the two groups; patients with RLS were significantly younger compared to those without RLS (p<0.05). The URR and Kt/V were not significantly different between the two groups (p>0.05). A comparison of other variables is depicted in Table 2.

**Table 2:** A comparison of RLS and non RLS groups in the study Abbreviations: URR - Urea Reduction Ratio; HD - Hemodialysis.

Variable	RLS present	RLS absent	P Value
M/F	33/34	112/100	0.610
Mean URR (%)	64.70 ±16.50	65.70 ±14.97	0.740
Mean Session Length (Hours)	3.88 ± 0.31	3.88 ± 0.37	0.842
Mean Kt/V	1.30 ± 0.20	1.30 ±0.18	0.649
Mean Duration on HD (Months)	30.88 ± 21.70	25.07 ± 22.97	0.018
Mean HD Sessions	261.22 ± 176.50	218.56 ± 191.67	0.029
Hemoglobin (g/L)	10.13 ± 1.53	9.92 ± 1.60	0.267
Mean Age (Years)	49.51 ± 18.47	54.55 ± 18.50	0.035
Mean Serum Albumin (g/L)	3.66 ± 0.38	3.55 ± 0.46	0.103

## Discussion

Restless legs syndrome is a common neurological disorder in dialysis patients. The prevalence varies greatly all over the world. The reported prevalence of RLS ranges from 20-73% in renal dialysis patients [2]. Some studies in Pakistan describe prevalence between 20 and 65%. The prevalence of the disorder in our dialysis patient population was 24%. The differences may be secondary to different patient characteristics, diagnostic criteria, and sample sizes.

The available literature does not describe any age group as a risk factor for the occurrence of RLS [1,2]. Though reported before in a few studies, the association with female gender is controversial [11]. We did not find any association between gender and RLS in our study. Patients with RLS were significantly younger than those without RLS (Table 2). This may be explained by a greater proportion of young patients in our study sample who have been on HD for a long time. Difficulty in finding a suitable donor, costs of transplantation, and, consequently, a greater duration on HD may be plausible explanations for this finding, which has not been observed in past studies.

The mechanism of RLS is poorly understood. Studies have identified a variety of both central and peripheral nervous system abnormalities in this disorder. There is no evidence of neurodegeneration as a mechanism. Some described associations include genetics (positive family history), iron deficiency, renal failure, spinal cord pathologies, neuropathies, and essential tremor [15-17].

Since we were studying RLS in the HD population and determining any associations with dialysis-related parameters, we tried to exclude confounding variables. All patients with the aforementioned associations to RLS were excluded. Anemia associated with iron deficiency has been reported as a predisposing factor for RLS in different patient populations [1,10,11]. Therefore we excluded patients with iron deficiency to make our findings more authentic with respect to dialysis parameters. Although the mean hemoglobin levels in both RLS and non-RLS patients were less than the specified targets for the HD population (11.5 g/L), patients were not iron deficient as evidenced by the normal mean ferritin and transferrin saturation [18]. Thus, we did not find an association between RLS and anemia in the absence of iron deficiency.

Dialysis quality is a predictor of mortality in dialysis patients [14]. Although numerous measures exist, URR and Kt/V are the most accurate and commonly employed. Several studies have shown that a URR above 65% or Kt/V of 1.2 is effective in improving a dialysis patient’s prognosis [14]. Dialysis adequacy takes into account the removal of urea from the blood after a session of hemodialysis. Kt/V is deemed more accurate. Past studies did not find an association between RLS and dialysis inadequacy. Our results are essentially the same in this regard as evidenced by Table 2. Past literature does not describe an association between dialysis frequency and RLS [1,15]. Our results reiterate the same. We must mention though, that because our center is a private institution, most patients undergo HD twice a week; this does have an impact on the dialysis adequacy but did not show an association with RLS.

Other important dialysis-related parameters include the dialysis session duration and total duration of HD. We did not find an association between RLS and the length of the sessions, which seems to be a consistent finding in almost all studies [1]. Some previous studies attributed increased HD duration as a risk factor for RLS [15]. We observed that patients with RLS were on a statistically significant longer duration of HD as compared to the non-RLS group (Table 2). This is also reinforced by the fact that the RLS group had a greater mean number of sessions as compared to the non-RLS group and the results were statistically significant (Table 2). It is well known that renal transplantation improves RLS symptoms while remaining on HD does not alleviate them in all patients. It may be that the chronic inflammatory state induced by hemodialysis affects the development of RLS, and a longer duration exposes to greater inflammation and risk of developing the disorder. Abnormalities of microvascular function in the legs have already been demonstrated in RLS patients, including altered leg intramuscular blood flow, peripheral hypoxia, and endothelial dysfunction [16,17].

## Conclusions

RLS is a common disorder in hemodialysis patients that results in a poor quality of life. We identified a prevalence of 24% in our large HD patient population, which is in accordance with the national data. A longer duration and greater number of HD sessions were significantly associated with RLS in our study. Dialysis inadequacy, however, does not seem to associate with the disorder. The pro-inflammatory nature of HD may have a role in the pathophysiology of RLS in HD patients and prolonged exposure may make them more prone to it.
